# Epidemiology of Methicillin-Resistant *Staphylococcus aureus* Diabetic Foot Infections in a Large Academic Hospital: Implications for Antimicrobial Stewardship

**DOI:** 10.1371/journal.pone.0161658

**Published:** 2016-08-24

**Authors:** Kelly R. Reveles, Bryson M. Duhon, Robert J. Moore, Elizabeth O. Hand, Crystal K. Howell

**Affiliations:** 1 College of Pharmacy, The University of Texas at Austin, Austin, Texas, United States of America; 2 Pharmacotherapy Education & Research Center, University of Texas Health Science Center San Antonio, San Antonio, Texas, United States of America; 3 University Health System, San Antonio, Texas, United States of America; Universitatsklinikum Munster, GERMANY

## Abstract

**Introduction:**

Diabetic foot infections (DFIs) are the leading cause of non-traumatic lower extremity amputations in the United States. Antimicrobials active against methicillin-resistant *Staphylococcus aureus* (MRSA) are recommended in patients with associated risk factors; however, limited data exist to support these recommendations. Due to the changing epidemiology of MRSA, and the consequences of unnecessary antibiotic therapy, guidance regarding the necessity of empirical MRSA coverage in DFIs is needed. We sought to 1) describe the prevalence of MRSA DFIs at our institution and compare to the proportion of patients who receive MRSA antibiotic coverage and 2) identify risk factors for MRSA DFI.

**Methods:**

This was a retrospective cohort study of all adult, culture-positive DFI patients managed at University Hospital, San Antonio, TX between January 1, 2010 and September 1, 2014. Patient eligibility included a principal ICD-9-CM discharge diagnosis code for foot infection and a secondary diagnosis of diabetes. The primary outcome was MRSA identified in the wound culture. Independent variables assessed included patient demographics, comorbidities, prior hospitalization, DFI therapies, prior antibiotics, prior MRSA infection, and laboratory values. Multivariable logistic regression was used to identify risk factors for MRSA DFI.

**Results:**

Overall, 318 patients met inclusion criteria. Patients were predominantly Hispanic (79%) and male (69%). Common comorbidities included hypertension (76%), dyslipidemia (52%), and obesity (49%). *S*. *aureus* was present in 46% of culture-positive DFIs (MRSA, 15%). A total of 273 patients (86%) received MRSA antibiotic coverage, resulting in 71% unnecessary use. Male gender (OR 3.09, 95% CI 1.37–7.99) and bone involvement (OR 1.93, 1.00–3.78) were found to be independent risk factors for MRSA DFI.

**Conclusions:**

Although MRSA was the causative pathogen in a small number of DFI, antibiotic coverage targeted against MRSA was unnecessarily high.

## Introduction

In 2014, the Centers for Disease Control and Prevention estimated that there were 29.1 million people living with diabetes in the United States (U.S.), representing 9.3% of the U.S. population [1]. Foot ulcers and subsequent infections are a serious, yet common, consequence of long-standing, uncontrolled diabetes. Diabetic foot infections (DFIs) are the leading cause of non-traumatic lower extremity amputations and result in approximately 66,000 amputations each year in the U.S. Additionally, the costs associated with DFIs are approximately $174 billion annually [1].

*Staphylococcus aureus* is a commonly reported pathogen among DFIs. This pathogen presents many treatment difficulties, particularly in the provision of appropriate empiric antimicrobial therapy. Approximately 40–50% of all *S*. *aureus* isolates exhibit methicillin resistance which confirms almost universal beta-lactam resistance. Recent treatment guidelines have recommended empiric anti-Staphylococcal coverage for all patients with a DFI [2]. The need for antimicrobials active against methicillin-resistant *S*. *aureus* (MRSA) is recommended in patients with risk factors associated with MRSA infections, specifically previous MRSA infection and high local prevalence of MRSA [2]; however, limited data exist to support these recommendations [3–5].

The rapid rise of antimicrobial resistance, specifically MRSA, during the first decade of the 21st century posed many problems for practitioners. However, recent reports have indicated a decrease in the prevalence of MRSA in certain disease states [6, 7]. Due to the changing epidemiology of MRSA, and the consequences of unnecessary antibiotic therapy, guidance regarding the necessity of empiric MRSA coverage in DFIs is needed.

The objectives of this study were to: 1) describe the prevalence of MRSA DFIs at a large academic teaching hospital and compare to the proportion of patients who receive MRSA antibiotic coverage and 2) identify risk factors for MRSA DFI.

## Methods

### Study design

This study was approved by the Institutional Review Boards at the University of Texas Health Science Center San Antonio and University Health System, San Antonio, TX. Both institutions waived the need for informed consent. This was a retrospective cohort study of all DFI patients managed at University Hospital, San Antonio, TX between January 1, 2010 and September 1, 2014. We included all hospitalized, adult patients (age 18–89 years) with a principal *International Classification of Diseases*, *9th Revision*, *Clinical Modification* (ICD-9-CM) code for foot infection and a secondary code for diabetes within the study period (Table 1). We limited our cohort to only those with at least one DFI pathogen identified through microbiological analysis. Patients meeting inclusion criteria were identified using an electronic search for ICD-9-CM codes using administrative records, followed by a manual chart review to confirm DFI diagnosis and to collect all other variables.

**Table 1:** ICD-9-CM codes for DFI, health outcomes, and comorbidities.

Diagnosis or procedure	ICD-9-CM code
Foot infection	
Gangrene	040.0; 440.24; 785.4 + (250.7 or 440.2X)
Osteomyelitis	730.07; 730.17; 730.27; 730.97
Ulcer	440.23; 707.1X
Cellulitis/abscess of foot	680.7; 682.7
Cellulitis/abscess of toe	681.10
Paronychia	681.11
Diabetes	250.00–250.93

### Study definitions

Patient demographic characteristics were identified at the time of the eligible hospital visit and included age, sex, self-reported race, and self-reported Hispanic ethnicity. Comorbidities were also assessed at the time of the eligible visit and included all Charlson comorbidities, as well as any infection with methicillin-susceptible *Staphylococcus aureus* (MSSA), MRSA, any Enterococcus species, or vancomycin-resistant enterococci (VRE) in the 30 days prior to the eligible visit. The following health care-associated variables were also collected: hospitalization for two or more days in the past 90 days, hospital length of stay, comorbidities, DFI therapies, duration of therapy, prior intravenous or oral antibiotics in the past 30 days, and chronic hemodialysis. Vital signs and laboratory values were collected on the day of DFI diagnosis if available. All antibiotics received in the hospital or prescribed for outpatient use following discharge were recorded. We defined MRSA therapy as receipt of any of the following antibiotics: vancomycin, daptomycin, linezolid, clindamycin, doxycycline, minocycline, tetracycline, and trimethoprim-sulfamethoxazole. DFI was classified by severity using a modified Infectious Diseases Society of America severity classification as outlined in the clinical practice guidelines [2]. A mild infection was defined as a local infection requiring oral antibiotics only. A moderate infection was defined as an infection of deeper tissues or bone involvement requiring intravenous antibiotics. Lastly, severe infections were those in which patients required intravenous antibiotics and met two or more systemic inflammatory response syndrome criteria. Bone involvement was defined as a diagnosis of osteomyelitis. Finally, all pathogens identified in the DFI wound culture were recorded. Pathogens were identified by Gram stain, biochemical testing, and the Vitek^®^ 2 System (bioMérieux, Inc.) in accordance with guidance from the Clinical Laboratory Standards Institute (CLSI). MRSA II CHROMagar^®^ (BD^™^) was used to screen all cultures for the presence of MRSA. The primary dependent variable was a positive MRSA culture.

### Statistical analysis

JMP 11.0^®^ (SAS Corp., Cary, NC) was used for all statistical analyses. We first described our patient population using medians and interquartile ranges for continuous variables and counts and percentages for categorical variables. We described the proportion of patients with MRSA DFI compared to other pathogens. These data were presented as counts and percentages and compared using the chi-square test. Next, we calculated the proportion of patients who received MRSA-targeted therapy. Finally, we identified independent predictors for MRSA DFI using a logistic regression model with MRSA as the dependent variable and all variables that were significant (p<0.05) in bivariable analyses (male sex, hypertension, prior MRSA, white blood cell count, severe DFI and bone involvement) as covariates.

## Results

### Patient characteristics

The limited dataset used to analyze study data can be found in the S1 File. Overall, 318 patients met inclusion criteria. Table 2 describes the patients’ baseline characteristics. Patients had a median (interquartile range) age of 52 (45–59) years and were predominately male (69%) and Hispanic (79%). The median (interquartile range) Charlson Comorbidity Score was 4 (3–6) and common comorbidities included: hypertension (76%), dyslipidemia (52%), obesity (49%), peripheral vascular disease (37%), and kidney disease (12%). Common previously identified MRSA risk factors were intravenous (15%) or oral antibiotics (43%) in the last 30 days and recent hospitalization (19%). Nearly one-quarter (24%) of patients’ infections were classified as severe and nearly half (48%) had bone involvement (i.e., osteomyelitis). A total of 123 (39%) patients received a lower-extremity amputation during hospitalization.

**Table 2:** Patient characteristics.

Characteristic	Overall (n = 318)	No MRSA (n = 271)	MRSA (n = 47)	P-value
Age (years), median (IQR)	52 (45–59)	53 (45–59)	49 (43–57)	0.0888
Male sex, n (%)	217 (69)	176 (66)	40 (85)	**0.0085**
White race	296 (94)	252 (94)	43 (94)	0.8778
Hispanic ethnicity	243 (79)	208 (80)	34 (76)	0.5285
BMI, median (IQR)	30 (25–34)	30 (26–35)	31 (25–33)	0.5305
Comorbidities, n (%)				
Peripheral neuropathy	224 (70)	188 (70)	35 (75)	0.5027
Diabetic retinopathy	43 (14)	35 (13)	7 (15)	0.7186
Dyslipidemia	164 (52)	139 (52)	25 (53)	0.8286
Hypertension	242 (76)	212 (79)	29 (62)	**0.0105**
Obesity	153 (49)	130 (49)	23 (49)	0.9751
Myocardial infarction	26 (8)	24 (9)	1 (2)	0.2343
Congestive heart failure	37 (12)	34 (13)	3 (6)	0.2211
Peripheral vascular disease	118 (37)	101 (38)	15 (32)	0.4421
Cerebrovascular disease	30 (9)	28 (10)	2 (4)	0.1862
Dementia	5 (2)	3 (1)	2 (4)	0.1103
Chronic obstructive pulmonary disease	6 (2)	5 (2)	1 (2)	0.8981
Peptic ulcer disease	6 (2)	5 (2)	1 (2)	0.8981
Moderate/severe kidney disease	39 (12)	32 (12)	7 (15)	0.5579
Cancer	8 (3)	8 (3)	0 (0)	0.3470
Mild liver disease	10 (3)	10 (4)	0 (0)	0.1800
Moderate/severe liver disease	4 (1)	3 (1)	1 (2)	0.5645
HIV/AIDS	2 (1)	2 (1)	0 (0)	0.5539
Charlson score, median (IQR)	4 (3–6)	4 (3–6)	4 (3–5)	0.9357
Prior infections, n (%)				
MSSA	20 (6)	15 (6)	5 (11)	0.1859
MRSA	24 (8)	17 (6)	7 (15)	**0.0398**
Enterococcus spp.	19 (6)	14 (5)	5 (11)	0.1461
VRE	2 (1)	2 (1)	0 (0)	0.5539
Health care association, n (%)				
Intravenous antibiotics in last 30 days	47 (15)	39 (15)	7 (15)	0.9357
Oral antibiotics in last 30 days	136 (43)	117 (44)	18 (38)	0.5194
Hospitalization ≥ 2 days in last 90 days	60 (19)	51 (19)	9 (19)	0.9665
Chronic hemodialysis	17 (5)	13 (5)	4 (9)	0.2993
Admission labs/vitals, median (IQR)				
Serum creatinine, mg/dL	1 (0.8–1.5)	1 (0.8–1.5)	1 (0.8–1.4)	0.6147
White blood cells, 10^9^/L	11 (8–14)	11 (8–14)	12 (9–15)	**0.0344**
Temperature, mmHg	98 (98–99)	98 (98–99)	98 (98–99)	0.8739
Heart rate, beats/min	88 (77–99)	88 (77–100)	87 (80–95)	0.2397
Respiratory rate, breaths/min	18 (18–20)	18 (18–20)	18 (18–20)	0.4806
C-reactive protein, mcg/dL	79 (28–162)	77 (24–164)	81 (61–147)	0.4795
Erythrocyte sedimentation rate, mm/hr	93 (60–109)	94 (64–110)	92 (38–107)	0.1375
Hemoglobin A1c, g/dL	10 (7–12)	10 (8–12)	9 (7–11)	0.3639
DFI severity, n (%)				
Mild	15 (5)	15 (5)	0 (0)	0.1004
Moderate	226 (71)	195 (73)	30 (64)	0.2851
Severe	76 (24)	59 (22)	17 (36)	**0.0464**
Bone involvement	153 (48)	123 (46)	29 (62)	**0.0431**

HIV/AIDS = human immunodeficiency virus; MSSA = methicillin-sensitive *Staphylococcus aureus*; MRSA = methicillin-sensitive *Staphylococcus aureus*; VRE = vancomycin-resistant *Enterococcus*; IQR = interquartile range; DFI = diabetic foot infection

Note: Bold indicates statistical significance

### Pathogens and antibiotic therapy

The most common DFI pathogens identified through microbiological analysis are provided in Table 3. *S*. *aureus* was the most common pathogen, representing 46% of culture-positive DFIs. Overall, only 15% were classified as MRSA. A total of 273 patients (86%) received MRSA antibiotic coverage, resulting in 71% unnecessary use. Vancomycin was the most commonly prescribed antibiotic for DFI, accounting for 78% of all antibiotics. Piperacillin/tazobactam was also prescribed frequently, with 70% of patients receiving this antibiotic. Other commonly prescribed antibiotics included: ciprofloxacin (15%), clindamycin (13%), and doxycycline (11%). Ceftriaxone (6%), ampicillin/sulbactam (3.8%), and amoxicillin/clavulanate (2.8%) were not commonly used.

**Table 3:** Causative pathogens among culture-positive DFI patients, n = 318.

Organism	n (%)^a^
*Staphylococcus aureus*	146 (46)
Penicillin-sensitive	15 (5)
Methicillin-sensitive	84 (27)
Methicillin-resistant	47 (15)
*Streptococcus* spp.	103 (32)
Group B *Streptococcus*	71 (22)
Coagulase-negative *Staphylococcus*^b^	58 (18)
*Enterococcus* spp.	64 (20)
*Pseudomonas aeruginosa*	25 (8)
Other Gram-negatives	53 (17)
Anaerobes	14 (4)

^a^Percentages combine to greater than 100% due to polymicrobial infections in some patients

^b^Coagulase-negative *Staphylococci* were not further differentiated by species

### Risk factors for MRSA DFI

In bivariable analyses, patients with MRSA differed significantly from those with any other pathogen with respect to several variables. Patients with MRSA were more often male (85% versus 66%, p = 0.0085), less likely to have hypertension (62% versus 79%, p = 0.0105), and were more likely to have been previously diagnosed with MRSA (15% versus 6%, p = 0.0398). Patients with MRSA were also more likely to have a severe infection (36% versus 22%, p = 0.0464), or bone involvement (62% versus 46%, p = 0.0431). Median white blood cell count was also higher in the MRSA group (12 x 10^9^/L versus 11 x 10^9^/L). In the multivariable analysis, only male gender (OR 3.09, 95% CI 1.37–7.99) and bone involvement (OR 1.93, 1.00–3.78) were found to be independent risk factors for MRSA DFI.

## Discussion

This study identified *S*. *aureus* as the most common pathogen among inpatients with DFI in a large academic teaching hospital; however, the rate of MRSA was low. This finding is of particular interest considering that nearly three-quarters of patients received anti-MRSA therapy. Furthermore, male sex and bone involvement were identified as independent risk factors for MRSA DFI.

*S*. *aureus* is the most common pathogen among skin and soft tissue infections (SSTIs), [8] though recent studies have demonstrated a decline in MRSA SSTIs in recent years [7]. The prevalence of MRSA DFI among inpatients ranges from approximately 5% to 20%, with less clear trends than non-DFI SSTIs [5]. Interestingly, prior studies have not demonstrated worse outcomes among DFI patients with MRSA compared to other pathogens [9–12].

Our study identified male sex and bone involvement as risk factors for DFI. To our knowledge, this is the first study to report male sex as a risk factor for MRSA specifically in DFI patients; however, one prior study found male sex to be associated with acquisition of MRSA in hospitals [13]. This might be attributed to the higher prevalence of MRSA risk factors among men as compared to women. Hartemann-Heurtier et al. [10] found osteomyelitis to be a risk factor for multidrug-resistant organisms in DFI. This might be due to poor penetration of antibiotics into the bone, which would select for resistant bacterial strains.

Other studies have noted the following risk factors for MRSA DFI: recent antibiotic use, previous hospitalization, extended duration of the foot wound, and nasal carriage of MRSA [10, 11, 14, 15]. The most commonly cited risk factor, as one might expect, is a history of MRSA infection. Prior MRSA infection and severe infection were statistically higher among patients presenting with MRSA DFI in bivariable analyses, though these factors did not remain statistically significant in multivariable models.

Knowledge of MRSA prevalence and identification of those patients most likely to be infected with MRSA could help guide clinician decision-making to use more aggressive therapies in those who need it most, while limiting aggressive therapy in low-risk patients. This would be especially important for those who participate in antimicrobial stewardship programs. The Centers for Disease Control and Prevention reported that as much as 50% of all antibiotic use is inappropriate. The improper use of antibiotics unnecessarily exposes the patient to potential complications of the therapy. Furthermore, the overuse of antibiotics drives antimicrobial resistance and is likely to increase the health care burden. We encourage facilities to closely monitor the prevalence of MRSA to help drive clinician decision-making in treating DFI. As unnecessarily aggressive therapy targeted against multidrug-resistant organisms has been associated with higher mortality in patients in other disease states, it is paramount to identify patients at high- and low-risk of MRSA DFI in order to provide tailored therapy [16].

This study has potential limitations. First, we utilized a retrospective cohort design that includes data collection from electronic medical records. Cohort studies might be subject to misclassification bias and confounding by unmeasured variables. Additionally, electronic medical data are created for the purpose of patient care, not for research, and might contain errors. Next, we utilized a single-center, inpatient, predominately Hispanic DFI population; therefore, results might not be generalizable to outpatients or populations with different demographics. Furthermore, prior hospitalization and antibiotic use were limited to the study hospital or as specifically noted in the medical record, which could potentially lead to misclassification bias of these MRSA risk factors. Physician preferences and other unmeasured factors, such as nasal MRSA carriage, may have influenced the decision to initiate one antimicrobial agent over another; however, we were unable to determine these associations with our study design. We were also unable to determine the importance of certain bacterial species, like Enterococci and Group B Streptoccoci, as this study was not designed to differentiate between contaminant and true DFI pathogen. Lastly, our sample size was relatively small which could have limited the power to detect differences among MRSA and non-MRSA DFI patients.

## Conclusions

Although MRSA was the causative pathogen in a small number of DFIs, antibiotic coverage targeted against MRSA was unnecessarily high. Our findings don’t support empiric use of anti-MRSA therapy in all DFI patients; however, larger epidemiological investigations are needed.

## Supporting Information

S1 File Limited data file. (XLSX)
